# Standardization of assay representation in the Ontology for Biomedical Investigations

**DOI:** 10.1093/database/baab040

**Published:** 2021-07-09

**Authors:** Randi Vita, Jie Zheng, Rebecca Jackson, Damion Dooley, James A Overton, Mark A Miller, Daniel C Berrios, Richard H Scheuermann, Yongqun He, Hande Küçük McGinty, Mathias Brochhausen, Aisyah Yu Lin, Sagar B Jain, Marcus C Chibucos, John Judkins, Michelle G Giglio, Irene Y Feng, Gully Burns, Matthew H Brush, Bjoern Peters, Christian J Stoeckert Jr

**Affiliations:** Center for Infectious Disease and Vaccine Research, La Jolla Institute for Immunology, 9420 Athena Circle, La Jolla, CA 92037, USA; Department of Genetics and Institute for Biomedical Informatics, Perelman School of Medicine, University of Pennsylvania, 3400 Civic Center Blvd, Philadelphia, PA 19104, USA; Center for Infectious Disease and Vaccine Research, La Jolla Institute for Immunology, 9420 Athena Circle, La Jolla, CA 92037, USA; Knocean Inc, Toronto, 105 Quebec Ave, ON M2P 2T3, Canada; Centre for Infectious Disease Genomics and One Health, Simon Fraser University, 8888 University Dr, Burnaby, BC V5A 1S6, Canada; Center for Infectious Disease and Vaccine Research, La Jolla Institute for Immunology, 9420 Athena Circle, La Jolla, CA 92037, USA; Knocean Inc, Toronto, 105 Quebec Ave, ON M2P 2T3, Canada; Institute for Biomedical Informatics, Perelman School of Medicine, University of Pennsylvania, 3400 Civic Center Blvd, Philadelphia, PA 19104, USA; USRA/NASA Ames Research Center, Building N-260, Moffett Field, CA 94305, USA; Center for Infectious Disease and Vaccine Research, La Jolla Institute for Immunology, 9420 Athena Circle, La Jolla, CA 92037, USA; Department of Informatics, J. Craig Venter Institute, 4120 Capricorn Ln, La Jolla, CA 92037, USA; Department of Pathology, University of California, 9500 Gilman Dr, San Diego, CA 92093, USA; Center for Computational Medicine and Bioinformatics, University of Michigan Medical School, 1500 E Medical Center Dr, Ann Arbor, MI 48109, USA; Department of Chemistry and Biochemistry, Ohio University, 1 Ohio University Drive, Athens, OH 45701, USA; Translational Research Institute, University of Arkansas for Medical Sciences, 4301 W Markham St, Little Rock, AR 72205, USA; National Center for Ontological Research, University at Buffalo, 126 Park Hall, Buffalo, NY 14260, USA; Department of Informatics, J. Craig Venter Institute, 4120 Capricorn Ln, La Jolla, CA 92037, USA; Institute for Genome Sciences, University of Maryland School of Medicine, 655 W Baltimore St, Baltimore, MD 21201, USA; Department of Biology, University of Pennsylvania, 3400 Civic Center Blvd, Philadelphia, PA 19104, USA; Institute for Genome Sciences, University of Maryland School of Medicine, 655 W Baltimore St, Baltimore, MD 21201, USA; Department of Psychology, University of Illinois Urbana-Champaign, 506 S. Wright St, Champaign, IL 61820, USA; Chan Zuckerberg Initiative, 801 Jefferson Ave, Redwood City, CA 94062, USA; Oregon Health and Science University, 3181 SW Sam Jackson Park Rd, Portland, OR 97239, USA; Center for Infectious Disease and Vaccine Research, La Jolla Institute for Immunology, 9420 Athena Circle, La Jolla, CA 92037, USA; Department of Medicine, Division of Infectious Diseases and Global Public Health, University of California, 9500 Gilman Drive, La Jolla, CA 92037, USA; Department of Genetics and Institute for Biomedical Informatics, Perelman School of Medicine, University of Pennsylvania, 3400 Civic Center Blvd, Philadelphia, PA 19104, USA

## Abstract

The Ontology for Biomedical Investigations (OBI) underwent a focused review of assay term annotations, logic and hierarchy with a goal to improve and standardize these terms. As a result, inconsistencies in W3C Web Ontology Language (OWL) expressions were identified and corrected, and additionally, standardized design patterns and a formalized template to maintain them were developed. We describe here this informative and productive process to describe the specific benefits and obstacles for OBI and the universal lessons for similar projects.

## Introduction

Experimental assays are central to biomedical research. They are used to generate data in a wide range of research domains, and the type of assay, inputs, devices used and protocols all impact the reliability, interpretation and reusability of the data generated. Describing assays using ontological realism provides a mechanism for capturing data provenance as well as facilitating the ability to integrate, search and compare the results of different studies and investigations (1). As a result, the need to formally represent assays drove the creation of the Ontology for Biomedical Investigations (OBI), and accordingly, assays are the core components of OBI.

OBI (http://obi-ontology.org/) has existed as a community volunteer project for >10 years, with the main objective of providing standardized ontology terms to model biomedical investigations (2). Its first official release was in 2009 (3). OBI covers all phases of the investigation process, including planning, execution, analysis and reporting. OBI contains terms related to the material entities and information that participate in investigations, as well as the roles and functions that material entities can play and that can be realized during the investigation. ‘Assay’ in OBI is defined as ‘a planned process with the objective to produce information about the material entity that is the evaluant, by physically examining it or its proxies’. In the latest OBI release (http://purl.obolibrary.org/obo/obi/2020-08-24/obi.owl), 897 terms out of a total of 2939 classes are assays or are assay related, such as the reagents and devices used in specific assay types.

The terms in OBI were added over the years by many different researchers working on projects with diverse needs and perspectives. Currently, terms are typically requested via a term tracker (https://github.com/obi-ontology/obi/issues) and vetted on weekly web-based OBI developer conference calls. This process has evolved as the ontology grew and as different contributors joined and left. The majority of this work was driven by specific projects needing investigation-related terms, resulting in a member of the project becoming active in OBI development for a limited period of time. Thus, many of the terms in OBI were added by project members by following their projects’ own personalized design patterns. This is typical of community-driven ontologies and is not necessarily bad, as OBI exists to serve all research communities and requires actual use cases to be of value and to test the logic behind the hierarchy. However, the result is a lack of uniformity of design patterns over the various portions of the ontology and presents challenges to developing continuity of design pattern development as contributors come and go.

During the generation of the assay terms, OBI developers identified that many assay terms shared common design patterns. Therefore, a Quick Term Template was initially employed to add sets of assay terms following the same pattern, using a tab-delimited format file (4). However, due to the needs of various applications and lack of easy-to-use tools, some assay terms were added to OBI that did not follow the shared pattern. Furthermore, different OWL expressions were used to represent the same pattern.

The uUse of the ontology has continued to grow and mature as the volunteer community maintaining OBI has also become more stable and experienced. More than 30 projects have contributed to OBI, and new projects continue to join (3). After OBI grew to include a substantial number of terms, it became apparent that a comprehensive review of the assay terms was warranted to promote further use and growth of OBI. The goals of this concentrated assay review were to compare existing design patterns, to identify and correct inconsistencies of OWL expressions among terms, to formalize a template to facilitate easier addition of new terms, to ensure that all assays were represented consistently in OWL and to perform a general philosophical review of the existing assay term logic and hierarchy. Thus, the specific targets of the review were the assay terms, as well as all of their attributes, including textual definition, axioms, examples, synonyms, etc. This was a very informative process that took place over several years and fostered much discussion. Here, we describe this process and relate its success in improving OBI. Our experiences were very positive, and we have begun to adopt the same review process in other areas of OBI. We advocate for developers of other ontologies to consider performing similar review and standardization activities.

## Materials and methods

### Identification of assay concepts

OBI developers retrieved all assay terms, along with their associated annotations and axioms from the OWL format ontology and placed them into a spreadsheet format file. The spreadsheet format was chosen to facilitate group review and easily identify discrepancies between rows and columns, especially for missing information.

The most common terms were identified among the components of logical axioms in the spreadsheet: ‘evaluant’, ‘analyte’, ‘input’, ‘output’, ‘objective’, ‘material processing technique’, ‘detection technique’, any ‘reagent’ or ‘device’ terms used in the assay and ‘parent class’. There was a great deal of variety in the properties utilized by any given term. The majority of assay terms, however, had a core set of six key annotation properties: ‘ontology id’, ‘label’, ‘definition’, ‘definition source’, ‘example of usage’ and ‘term editor’. Some terms also contained ‘alternative term’, particularly for terms that may have different meanings across different communities, or ‘editor note’, for those terms that may still be in process or if they involved a great deal of discussion. We also included ‘has curation status’ as a required annotation to be made consistent as part of this process. All terms and axioms were represented in separate columns of the spreadsheet and targeted for further review. Some example terms as they looked at the start of this process are shown in Table 1.

**Table 1.** Example assay terms and their annotations and the start of the assay review process

rdfs:label	ChIP-seq assay	in-situ hybridization assay	96-well neutralization assay	viral hemagglutination assay	DNase I hypersensitive sites sequencing assay
ID	OBI:0000716	OBI:0001686	OBI:0000865	OBI:0000871	OBI:0001853
has curation status	ready for release	ready for release	metadata complete	metadata complete	metadata complete
parent class	ChIP assay	assay	serum neutralization of viral infectivity assay	analyte assay	DNA sequencing
detection technique	DNA sequencing				DNA sequencing
evaluant					deoxyribonucleic acid
measurand				Viruses	
analyte					
device	DNA sequencer		multi-well plate		DNA sequencer
reagent					
input		labeled nucleic acid extract			deoxyribonucleic acid
output	information content entity			measurement datum	DNA sequence data
objective		biological feature identification objective			protein and DNA interaction identification objective
material processing technique		nucleic acid hybridization		induced hemagglutination	library preparation, non-specific enzymatic cleavage
definition	An assay in which chromatin is immunoprecipitated and subsequently analyzed using a DNA sequencing step to identify which parts of DNA are part of the isolated chromatin	An assay that localizes a specific DNA or RNA sequence within a portion or section of tissue using artificially induced nucleic hybridization.	A serum neutralization of viral infectivity assay which is performed in a 96-well plate.	An assay that quantifies viruses by their hemagglutination activity.	An assay that identifies the location of regulatory regions, based on the genome-wide sequencing of regions super sensitive to cleavage by DNase I.
definition source	adapted from Wikipedia	PMID:9021518		WEB: http://en.wikipedia.org/wiki/Hemagglutination_assay	http://en.wikipedia.org/wiki/DNase-Seq
term editor	Philippe Rocca-Serra, Bjoern Peters	PERSON: Philippe Rocca-Serra; Marcus Chibucos	person: Bjoern Peters person: Melanie Courtot	person: Bjoern Peters person: Melanie Courtot person: Randi Vita	Person: Venkat Malladi, Chris Stoeckert, Jie Zheng
alternative term	chromatin immunoprecipitation sequencing assay	ISH	microneutralization assay	viral haemagglutinin assay HIHA	
example of usage	PMID: 19275939 ChIP-seq: using high-throughput sequencing to discover protein-DNA interactions. Schmidt D, Wilson MD, Spyrou C, Brown GD, Hadfield J, Odom DT. Methods. 2009 Jul; 48 (3): 240–8. Epub 2009 Mar 9.	Use of in situ hybridization to examine gene expression in the embryonic, neonatal, and adult urogenital system. PMID:22639265		Determining the viral titer of a virus infected human by measuring the presence of hemagglutination when dilutions of serum samples are added to a known quantity of red blood cells.	Sabo, et al. Discovery of functional noncoding elements by digital analysis of chromatin structure. Proc Natl Acad Sci U S A. 2004 Nov 30; 101(48): 16837–42. [PMID:15550541]
editor note			MC: 20100217: microneutralization is used by the influenza community, to refer to a nutralizationa ssay at a smaller scale. However smaller is difficult to define accurately, and we therefore chose a label being more specific.		

### Review of assay terms

Once all needed information was retrieved, we set out to systematically review all terms in depth and apply edits as needed. Weekly web calls focused on this activity for 30 weeks and additionally, a face-to-face meeting was held with this assay review as the primary agenda item. Approximately 20 active OBI developers were involved at the face-to-face meeting. OBI development tracker items (https://github.com/obi-ontology/obi/issues) and/or group emails were used to reach OBI contributors not present on calls or at the in-person meeting for their input on term reviews and editing. Related tracker items are shown in Supplementary Table S1.

Discussions related to all aspects of assay terms were documented in weekly agenda documents in the form of Google docs, by tracker items, and OBI developer group emails. Every row and column of the sheet of assays was reviewed for format, accuracy, spelling, missing data and inconsistencies.

We focused first on studying and discussing the set of existing design patterns used by different assay types to improve and standardize these design patterns to be consistent and also to accommodate the needs of the vast majority of assay terms. After we finalized the design patterns, we then reviewed the OWL expressions used to represent the patterns, with attention paid to any inconsistent implementation. Missing axioms and annotations were also identified and remedied. Additionally, annotations associated with assay terms were also reviewed and improved, such as adding missing ‘example of usage’ or ‘definition source’ and making the wording of ‘definition’ more uniform, precise and complete across all assays. In some cases, new annotations were added as needed to further clarify the intended meaning of a term.

For example, the ‘measurand role’ was added to specify what was being measured in assays, having the objective to measure the magnitude/concentration/amount of something that is the measurand.

The editing of assay terms was performed as a large group, by select subject matter subgroups and by individual editors. Active editing was performed on calls, at the face-to-face meeting and offline by individuals tasked with specific terms, sets of similar terms and/or particular attributes. For example, a contributor with subject matter expertise in genetics was assigned to review all polymerase chain reaction (PCR)–related assay terms. Similarly, all textual definitions were edited by a single person for spelling and grammar. After editing offline, each individual or subgroup presented their findings and edits for discussion with the larger OBI developer community. If certain problems required further research and/or discussion, we reached out to appropriate experts and/or the original term creator or submitter for resolution.

In addition to more scientific and controversial discussions related to assay terms, we performed basic level ‘cleaning’ of terms in the form of spelling and grammar corrections, as well as ensuring that each term was complete as far as the core set of six properties. This was largely completed by individual developers being assigned to a specific attribute to manage. In some cases, this process was as simple as adding capitalization. In others, it required in-depth research, for example, to identify and add relevant ‘example of usage’ or ‘definition’ annotations when none were present. This process, thus led to the need to identify subject matter experts (SMEs). In order for an ontology to be accepted by the scientific public as the resource for a given field, SMEs are a requirement. Because OBI is the cross-disciplinary standard for representing biomedical investigations, and as such, covers diverse fields of research, editing assay types requires a wide range of expertise. Consequently, not all currently active OBI developers at any given time will have expertise regarding any given assay term present in OBI. The existing assay types were assigned to the most suitable currently active developers at the time of the assay review, based upon their scientific background.

### Implementation and maintenance of assay terms

As part of the axiom review, it became clear that an automated template to maintain these term design patterns was necessary. ROBOT (a recursive acronym for “ROBOT is an OBO Tool”) is an open source tool for automating ontology development tasks that allows users to generate ontology terms with shared common design patterns automatically in tabular templates (5). We employed the ROBOT tool to apply the efforts of this project back into OBI and to maintain consistency going forward. The ontology editor Protege was used to review the OWL file generated by ROBOT with the Hermit reasoner to confirm that the inferred hierarchy of assay terms was as expected (6).

## Results

Some aspects of the approach we took for the OBI assay term review generated a significant amount of discussion due to disagreement among the developers. The most contentious aspects were the axioms that should be included in a common assay template. Axioms are the way logical definitions of ontology terms are represented in OWL. These computer-interpretable definitions are critical to the value of an ontology. They represent the relationships between terms and provide the ability to reason across the ontology. The axioms of assay terms at the outset of this review were highly variable, with some only having a single is_a relationship with its parent term, while others were quite complex defining ‘input’, ‘output’ and ‘device’, as well as ‘evaluant’. A primary goal of this project was to establish a standard axiomatic design pattern for all assay terms, so a great deal of attention was paid to this aspect of the review. Here we present the most interesting aspects of the assay term review process used to establish a stable assay design pattern.

**Figure 1.**
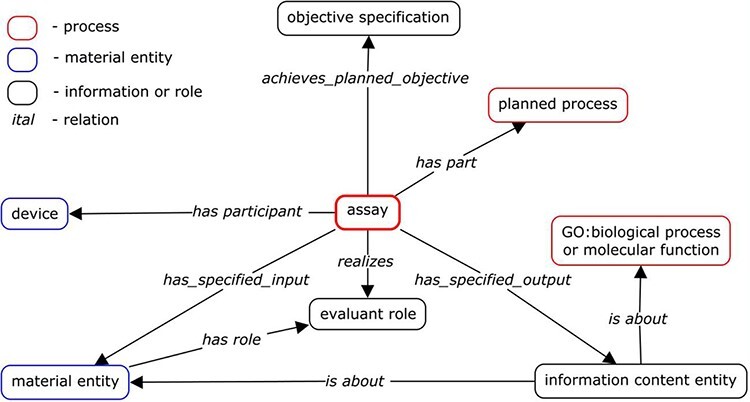
Original assay design pattern (3).

Extensive discussion among OBI developers determined the main axiom components needed to describe any assay term. Some developers wanted to be more inclusive and include a larger number of axioms, while others wanted to create the most simplified template possible that could handle the majority of existing assay terms. On review, we found the main components of many assay terms in OBI to be ‘output’ and ‘inputs’ such as ‘evaluant’, ‘reagent’, ‘analyte’ and ‘device’. Sometimes ‘objective’ was also used. Axioms that were sometimes used, but did not make it into the standardized template were ‘target entity’ and ‘material processing technique’, as these did not seem to be relevant to a significant number of assays, despite being important for some assays. Prior to this assay review, OBI’s general design pattern existed as shown in Figure 1, illustrating how these components worked together to form a typical assay.

### Input and output

Assays are modeled to have materials (‘material entity’) that act as ‘input’ to the experimental assay and that have specified roles within the assay, such as the role ‘evaluant’. The ‘output’ of an assay is the data generated by the assay. We specified the relationship between the inputs and the outputs and the roles they play using logical axioms, for example, ‘has_specified_output some (measurement datum)’. All assays were then reviewed to determine if they did or should specify ‘input’ and ‘output’, as well as what roles each input should play. An important aspect of our process was testing if all axioms specified for a given assay type held for all instances of that type. For example, the assay type ‘enzyme-linked immunosorbent assay’ (ELISA) may be used in one scenario to measure antibody binding to a protein, but protein cannot be stated to be an ‘input’ for all ELISA assays, as another equally valid scenario is the use of an ELISA to measure antibody binding to a peptide, rather than to a protein. This illustrates the need for SMEs when formulating terms in OBI.

### Evaluant

We defined ‘evaluant’ as ‘A role that inheres in a material entity that is realized in an assay in which data is generated about the bearer of the evaluant role'. An example of the ‘evaluant role’ is ‘blood serum specimen’ for ‘measuring glucose concentration in blood serum assay’, as this assay is intended to always generate data about a blood specimen. The use of the ‘evaluant role’ in assay axioms was variable among assays, being used by only 29 assay terms. Many assay terms used this role in the following format: ‘has_specified_input some (“X” and (has_role some “evaluant role”))’. The use of ‘evaluant role’ in general and with each assay being reviewed was discussed and edited as needed.

### Analyte

‘Analyte role’ is defined in OBI as ‘A measurand role borne by a molecular entity or an atom and realized in an analyte assay which achieves the objective to measure the magnitude/concentration/amount of the analyte in the entity bearing evaluant role'. ‘Analytes’ are used to group a large branch of OBI assays deemed ‘analyte assay’ that are defined as ‘An assay with the objective to capture information about the presence, concentration, or amount of an analyte in an evaluant’. This is a useful distinction because it allows users of the ontology to find all assays having the same ‘analyte’ and to identify differences between similar assays having different ‘analytes’. Many children of this branch do not specify an input that plays the ‘analyte role’, although one is implied in that all ‘analyte assays’ result in information regarding an ‘analyte role’ (achieves_planned_objective some analyte measurement objective). The use of the role of ‘analyte’ by all analyte assays was reviewed and edited as part of this process whenever a specific ‘analyte’ could be identified and agreed upon. The particular ‘molecular entity’ or ‘atom’ relevant to a given assay was assigned as the ‘analyte’. If an assay was understood to require an ‘analyte’, but could not be inferred from its axioms to be a child of ‘analyte assay’, it was reviewed to determine if the assay did actually require an ‘analyte’ and/or if the assay axioms required edits to ensure it would be inferred as an ‘analyte assay’.

### Reagent

The role of ‘reagent’ is played by ‘material entities’ that are not being evaluated by the assay, but are still important to the ability to perform the assay. Assays may also specify one or more ‘reagents’ whenever they are always used by a given assay type, for example, ‘chromium release assay’ will always utilize the ‘reagent’ of ‘chromium-51’ as this is integral to this assay type. Axioms involving ‘reagents’ were also evaluated as part of this overall process and edited as needed.

### Device

Four hundred and seventy-six ‘device’ terms are present in OBI. They are infrequently specified in assay axioms simply due to the possibility that many assays may be performed using different kinds of ‘devices’. For example, a ‘cell proliferation assay’ can be performed using a ‘cytometer’ when simply counting the cells, but can also be performed using a ‘radiation measurement device’ when cells are radiolabeled. However, ‘device’ terms are present in OBI because they can be useful for specific projects and 78 assays are stated to use specific ‘devices’. For example, a ‘flow cytometry assay’ is always performed using a ‘flow cytometer’. Many different specific flow cytometers exist as children of ‘flow cytometer’ for cases where one may want to describe the use of a particular brand or type of flow cytometer, such as ‘FACS Canto’. ‘Devices’ are modeled as ‘(has_specified_input some X) and (realizes some (function and (“inheres in” some X)))’.

### Objective

The ‘objective’ of an assay is defined as ‘an objective specification to determine a specified type of information about an evaluated entity (the material entity bearing evaluant role)'. Fifty-three assays specified the ‘objective’ that the assay was intended to achieve at the start of our review. Some assays modeled the ‘objective’ as using the class axiom ‘achieves_specified_objective some “X”’ while others stated what the resulting assay-generated data are about using ‘has_specified_output some (information content entity and is about some biological_process)’, or some assays specified both. This was largely due to who submitted the assay term, rather than a philosophical difference. However, it is possible for one to use the same assay with very different ‘objectives’. For example, an ELISA may be performed with the purpose to determine if a serum sample from dengue-infected subjects contains antibodies binding to a specific dengue protein. Alternatively, an ELISA may be performed to determine if a sample of T cells are producing interleukin-2. Collectively, it could be said that the ‘objective’ of both scenarios was to determine if a chemiluminescent reaction took place, which does not convey the true ‘objectives’ of researchers performing either assay. ‘Objective’ is currently mainly used for automatic classifications, where a variety of different assays that achieve the same ‘objective’ can be grouped together. For example, both ‘transcription profiling by RT-PCR assay’ and ‘transcription profiling by array assay’ achieve the ‘objective’ of ‘transcription profiling identification’.

### Design pattern

**Figure 2.**
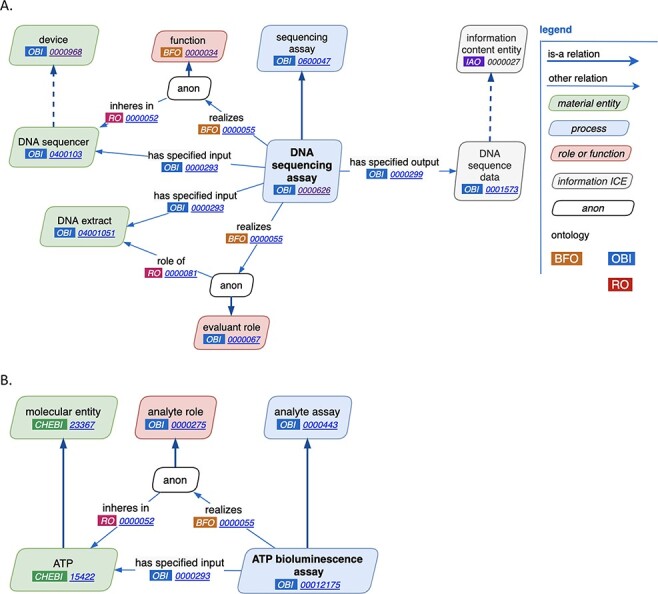
New assay patterns. (A). The general assay pattern logically connects the most commonly needed assay annotations. (B). The analyte assay pattern is distinguished by always including a specific ‘material entity’ playing the ‘analyte’ role. Note that ‘anon’ stands for ‘anonymous node’, which represents a multi-component axiom that a given class is a subclass of.

Through close manual inspection of each of the reviewed features of assays as discussed above, two new major design patterns were established, one for general assays and one for analyte assays. The general assay design pattern is shown in Figure 2A. Highlights of this pattern are the reuse of terms from other ontologies, the consistent use of annotations across a wide variety of assay types and comprehensive use of axioms. For example, in the ‘DNA sequencing assay’, a ‘DNA sequencer’ has the ‘device’, ‘DNA extract’ plays the ‘evaluant’ role and the ‘output’ of the assay is ‘DNA sequence data’. Figure 2B demonstrates an example analyte assay, ‘ATP bioluminescence assay’ where ATP is the analyte.

### ROBOT template

Early on in the review process, we realized the need for a systematic process to maintain the standardized design patterns going forward. Thus, we took advantage of the ROBOT ontology tool (5) and transitioned the spreadsheet used for manual assay review directly into a ROBOT template for automated ontology building. This was easily done, as ROBOT utilizes a spreadsheet format with annotations and axioms supplied in separate columns. All that was needed was the addition of a secondary header column to specify the mapping of the column contents to either an annotation or axiom, as the current assay template shown in Supplementary Table S2. The ROBOT template was then used to facilitate editing of existing terms and, going forward, to simplify the creation of new assay terms. New users of OBI can now add their terms to the template, with all the needed annotations and axioms presented in the universally understood spreadsheet format. This has resulted in more complete new term requests and has sped up the vetting process.

Once ROBOT was adopted, the OBI release process was updated to take advantage of its features. The OBI release process now uses ROBOT pervasively—combining import files and template modules, reasoning and automated quality control. Being a completely volunteer effort, this new release process is quite an advantage, allowing faster creation of new terms, now that less technical developers can perform a release with confidence. The result is a faster, more consistent release process that could be performed by a wider number of OBI developers and represents an unexpected benefit of the review process.

The guidelines for new OBI terms can be found at http://obi-ontology.org/page/OBI_term_guidelines, and the growing library of term templates can be found at https://github.com/obi-ontology/obi/tree/master/src/ontology/templates.

## Discussion

Our OBI assay term review process was an open, collaborative effort shared among many volunteer developers, keeping to the spirit of the OBO Foundry (7). As a result of this assay review process, existing OBI assay terms were improved and made more consistent, a new process for editing and adding terms was adopted (ROBOT), and furthermore, improvements were made to the release process and guidelines for the creation of new terms. Both the ontology terms and the OBI community were improved through this collaborative community effort, which made developers more aware of terms outside their area of expertise and gave them a better understanding of assay terms as a whole. By implementing standardized assay patterns, we were able to adopt the standardized ROBOT assay template for use going forward, which greatly improved the speed of new term requests and build cycles, making the ontology more responsive to its users. This template is easy to read by new users of OBI as it exists in a spreadsheet format. It also makes assay term requirements transparent. The experience of new users or first-time term requests is especially improved by the ability to view similar terms that follow a standard pattern in the template. This directs new users toward the expectations for annotations and axioms, making new term requests easier and simpler for the creator. The time needed to make new term requests and to incorporate those changes into OBI has drastically changed via the new release process, which is accessible to a wider number of developers. This example of ROBOT usage is illustrative of the benefits of adopting this free tool, both for the existing ontology developers and for new users.

We hope that the description of this process is helpful to other community ontology efforts and potential OBI users. We would have benefited from performing this review earlier in the life course of OBI, so that more new terms would have been added in a consistent manner; however, it is difficult to foresee such future needs, especially with a volunteer project. It was also difficult to make the time for such a thorough review when the participants are volunteers with many other responsibilities. Whenever possible, establishing design patterns early on during ontology development and adopting tools that enforce the accepted patterns are highly recommended. Additionally, because this was such a useful experience, we have since applied the same process to other groups of OBI terms that share similar patterns, such as ‘study design’ and ‘device’, and plan to do the same for ‘material processing’ and ‘data transformation’. As with the assay term review, the main obstacle will be setting aside the time needed to tackle each set of terms while maintaining the basic needs of the ontology. Increased funding for ontologies could mitigate these barriers to quality ontology development.

## Supplementary Material

baab040_Supp
